# Remarks, Gleanings, and Extracts

**Published:** 1874-04

**Authors:** T. Curtis Smith

**Affiliations:** Ohio


### REMARKS, GLEANINGS AND EXTRACTS

BY T. CURTIS SMITH, M.D., OF OHIO.

*Laws of Congenital Syphilis.*—In an address by Dr. E. Kennedy, President, Dublin Obstet. Soc., published in the *Dublin Med. Jour.*, January, 1874, the following propositions are laid down:

I have satisfied myself that syphilis is *par excellence* an hereditary disease.

That it descends from the father and mother through the tainted or poisoned ovum in both.

That its transmission through the circulation of the mother by taint in the course of the developement of the fœtus is possible.

That it may be transmitted in the secondary and tertiary stages, not in the primary, unless contract *in transitu* at birth.

That it may be transmitted in these stages by either parent without the contamination of the other.

That it may be transmitted when the disease, although latent, evinces no evidence of its existence beyond the taint in the germ.

That there is a tendency in time for the taint to wear out, or the poison to become weaker in both parents.

That the life of the fœtus and the period of gestation become prolonged from month to month in succeeding gestations, until at length a living child may be born, perhaps in the eight or ninth pregnancy. In such cases the poison is usually latent in the parent.

That syphilis is an occasional cause of barrenness in both parents, but more markedly in the male.

That generally it is not productive of interuptions to impregnation.

That the disease is under the influence of treatment in both parents.

That the proper time for treatment is before impregnation, and sufficiently long before to allow of the eradication of the poison, and a complete recuperative action being established and confined in the system.

That mercury, properly and sufficiently administered, followed by iodines and other alteratives, can eradicate the poison in one or both parents.

That its administration to afford the greatest security, ought to be extended to both, although evidence of the poison be perceptible only in one.

That as the effect of mercurial treatment is so disappointing when administered to the female, in the progress of pregnancy, it should not then be had recourse to, unless the state of the mother herself demand it; as, for instance, on the occurrence of a primary chancre or of urgent secondary symptoms.—*Med. and Surg. Reporter*, March 14th, 1874.

[We believe the above propositions so accurate, practical and full of common sense, and that careful observation will fully confirm their entire corretness, that we heartily commend their careful study and consideration to all our readers.]

*The Local Treatment of Cavities in the Lungs.*—The *Medical Times and Gazette*, Feb. 15, 1874, states that a novel method of treating pulmonary cavities in phthisis and dilatation of the bronchi was lately submitted by Professor Mosler, of Griefswald, to the notice of the members of the German Association, at their annual meeting at Wiesbaden. It consists in injecting certain drugs through the wall of the chest into superficial caverns, and leaving the canula in, so as to repeat the operation frequently at discretion. Mosler went even farther; he made an incision into the wall of the cavity, inserted a silver tube or elastic catheter, and succeeded in drawing away the secretion, and in disinfecting the pyogenic walls by means of a weak carbolic acid lotion. No difficulty was experienced in the operation, and the condition of the patient was improved; the cough became less troublesome, and the febrile symptoms apparently moderated.

Mosler's patient was still under treatment at the time of his communication, so that it was impossible to tell what the final result would be, and whether granulation and cicatrization would ensue. One point seemed settled, as far as it could be by experiments, and this was that the local treatment of pulmonary cavities is undoubtedly practicable, and that the lung is more tolerant of external interference than has generally been believed. At the same time, the risk of pneumo-thorax, hæmorrhage and pyæmia must not be forgotten.—*Boston Medical and Surgical Journal.*

*Ovarian Hernia.*—Dr. Benj. McCleur, of Dubuque, Iowa, relates in the *Amer. Jour. Obstet.*, a case of this rare difficulty in which he removed. The tumor was "soft, oval, about 1 x 1½ inches in diameter, occupying a position just below pouparts ligament, in the left groin." It usually gave no annoyance except during menstruation, when there was a pulling sensation as though connected with some of the internal organs. Later the tumor became very troublesome and was removed after which recovery was perfect. The specimen removed was examined microscopically by Dr. Paul F. Munde, who pronounced it "an ovary degenerated by cystic disease."

Of this class of cases there are but thirty-eight on record, nine of these were femoral, this last making ten femoral and adding one more to the entire list, making thirty-nine cases.

*Epilepsy Caused by a Needle in the Ear.*—Dr. R. S. Goodwin, of Thomaston, Conn., in the *Philadelphia Reporter*, relates the case of a young man who was cleaning his ear with a broken needle, which slipped from his fingers "into the meatus out of sight." He failed in his efforts to remove it, but felt no inconvenience from it for several months, except occasional slight pains in the aural region. He was then seized with a severe epileptic paroxysm, which recurred several different times. These however yielded to potass. brom. and argenti. nitr. Several months later the needle made its appearance at the upper and back part of the neck, from whence it was extracted. That the needle entered the ear was attested by different persons. The patient had never been subject to epileptic seizenes before, nor have they recurred since.

*Treatment of Enuresis.*—By Dr. Buyelmann, of Cologne.—The author was induced by an article in the Berlin *Klin. Wochenshrift*, 1871, No. vi., to try the syrup ferri iodidi in a severe case of incontinentia urinæ. The patient was a young girl, thirteen years old, of nervous temperament, and anæmic. The principal complaint was the incontinence of urine, so severe as to prevent her from walking any distance from her home without wetting herself. In addition to generous diet, she took, for three weeks, syrup ferri iodidi, seven grammes ad aquæ, syrup simp., aa, fifty grammes; a teaspoonful every two hours. After a week's treatment, there was a marked improvement, and, in two weeks more, she was discharged well.—*Berlin Klin. Wochenshrift.*

*Congenital Syphilis in Six Successive Infants.*—Dr. Wm. T. Taylor, of Philadelphia, Pa., in the *Amer. Jour. Obstet.*, February, 1874, relates an instance in which this disease affected the fœtus or infant in six successive pregnancies, in the first four the delivery was premature, and in the last two the infants though born at term soon manifested evidence of congenital syphilis, both dying early from attacks of diarrhœa coming on during the prevalence of syphilitic symptoms. The mother "is perfectly heatlhy" and it is probable that the fault is on the paternal side.

[This is one instance confirmatory of the rules laid down by Dr. Kennedy, in the foregoing article.]

*Lotion for Fœtid Feet.*—The *Union Médicale*, January 27, recommends permanganate of potash, fifteen parts, distilled water, 1000 parts, for this complaint. The feet are to be washed twice a day with the lotion, and, having been carefully dried, are to be powdered either with potato-starch or lycopodium.—*Boston Medical and Surgical Journal.*

*Silicate of Soda in Vesical Disease.*—Dr. M. Dubrenil recommends the injection of this salt to prevent decomposition of urine in agravated cases of vesical disease. Experiments prove that the salts of soda are antiputrid. One gramme of silicate of soda will prevent the decomposition of 100 grammes of urea for an indefinite time, and will also prevent its transformation into carb. ammonia. In a case of vesical disease from enlarged prostate, a solution of 4 grammes in 150 grammes of water was injected, with the effect of restoring the urine to its normal condition.—*Gaz. Med.—N. Y. Medical Journal,* January, 1874.

*Removal of Five Inches of the Sciatic Nerve.*—At the Roosevelt Hospital, a female patient was recently admitted on account of a tumor, which was situated in the posterior part of the left thigh. When cut down upon, the growth was found to be intimately connected with the sciatic nerve, and to such an extent as to preclude all possibility of its extirpation without completely removing a large portion of the nerve. Accordingly, about five inches of the nerve were removed. The patient was discharged from the hospital within three weeks, with but slight impairment of motion and sensation.—*N. Y. Medical Record.—Boston Med. and Surg. Jour.*

*Mass of Scybala Mistaken for an Ovarian Tumor.*—Dr. John Hall Davis reports a case in which a mass of scybala, exceeding in bulk a child's head at mature delivery, and giving rise to menorrhagia, occupied the left iliac fossa, and had been mistaken by a physician for an ovarian tumor. Dr. Davis appears to have hit at once upon a correct diagnosis by simply pressing upon the tumor, and thus demonstrating that the surface of the mass retained the impression of the fingers.—*Obstetrical Journal,* Jan., 1874.—*Boston Medical and Surgical Journal.*

*First Carotid Ligation in the West.*—Dr. Wm. W. Awl, at Clear Creek, near Lancaster, Ohio, ligated the carotid artery early in March, 1872, in the removal of an immense tumor from the side of the face and neck of a little girl, aged 12 or 13 years. The patient recovered from the operation. This is supposed to be the first instance of carotid ligation, west of the mountains in the United States, and the account was published in the *Western Medical and Physical Journal, Oct.,* 1827.—Trans. Ohio State Med. Soc. 1859.

*Inoffensive Sponge-Tents.*—Lawson Tait, F. R. C. S., in the *Medical Times and Gazette* of January 10th, recommends the use of a five per cent. solution of oil of cloves in the preparation of sponge-tents, as a means of rendering them inoffensive and obviating the danger of septic peritonitis from their use. He finds a tent thus prepared perfectly free from the usual disagreeable odor after remaining twenty-four hours in the uterus.—*N. Y. Medical Journal.*

